# Western oropharyngeal and gut microbial profiles are associated with allergic conditions in Chinese immigrant children

**DOI:** 10.1016/j.waojou.2019.100051

**Published:** 2019-08-09

**Authors:** Jing Guo, Quanjun Lv, Amir Ariff, Xiaoping Zhang, Christopher S. Peacock, Yong Song, Xiajie Wen, Aarti Saiganesh, Phillip E. Melton, Gary A. Dykes, Eric K. Moses, Peter N. LE Souëf, Fengmin Lu, Guicheng Zhang

**Affiliations:** a School of Public Health, Curtin University of Technology, Perth, WA, Australia; b Centre for Genetic Origins of Health and Disease, Faculty of Health Sciences, Curtin University of Technology, Faculty of Health and Medical Sciences, The University of Western Australia, Royal Perth Hospital Medical Research Foundation, Perth, WA, Australia; c Department of Nutrition and Food Hygiene, College of Public Health, Zhengzhou University, Zhengzhou, Henan, China; d China National Bamboo Research Centre, Key Laboratory of Resources and Utilization of Bamboo of State Forestry Administration, Hangzhou, Zhejiang, China; e Marshall Centre for Infectious Disease, School of Pathology and Laboratory Medicine, The University of Western Australia, Perth, WA, Australia; f Telethon Kids Institute, The University of Western Australia, Perth, WA, Australia; g Division of Cardiovascular and Respiratory Sciences, The University of Western Australia, Perth, WA, Australia; h School of Pharmacy and Biomedical Sciences, Faculty of Health Sciences, Curtin University of Technology, Perth, WA, Australia; i Department of Microbiology & Infectious Disease Centre, School of Basic Medical Sciences, Peking University Health Science Centre, Beijing, China

**Keywords:** Microbiome, Immigration, Western environment, Atopy, Allergy, AC, Australian Chinese, CC, China-Born Chinese, rRNA, ribosomal RNA, OP, oropharyngeal, SPT, skin prick test, BMI, body mass index, LDA, The linear discriminant analysis, LEfSe, The linear discriminant analysis effect size, PICRUSt, Phylogenetic Investigation of Communities by Reconstruction of Unobserved States, KEGG, Kyoto Encyclopaedia of Genes and Genomes, FDR, false discovery rate

## Abstract

**Background:**

The allergy epidemic resulting from western environment/lifestyles is potentially due to modifications of the human microbiome. Therefore, it is of interest to study immigrants living in a western environment as well as their counterparts in the country of origin to understand differences in their microbiomes and health status.

**Methods:**

We investigated 58 Australian Chinese (AC) children from Perth, Western Australia as well as 63 Chinese-born Chinese (CC) children from a city in China. Oropharyngeal (OP) and fecal samples were collected. To assess the microbiomes, 16s ribosomal RNA (rRNA) sequencing for variable regions V3 and V4 was used. Skin prick tests (SPT) were performed to measure the children's atopic status. Information on food allergy and wheezing were acquired from a questionnaire.

**Results:**

AC children had more allergic conditions than CC children. The alpha diversity (mean species diversity) of both OP and gut microbiome was lower in AC children compared to CC children for richness estimate (Chao1), while diversity evenness (Shannon index) was higher. The beta diversity (community similarity) displayed a distinct separation of the OP and gut microbiota between AC and CC children. An apparent difference in microbial abundance was observed for many bacteria. In AC children, we sought to establish consistent trends in bacterial relative abundance that are either higher or lower in AC versus CC children and higher or lower in children with allergy versus those without allergy. The majority of OP taxa showed a consistent trend while the majority of fecal taxa showed a contrasting trend.

**Conclusion:**

Distinct differences in microbiome compositions were found in both oropharyngeal and fecal samples of AC and CC children. The association of the OP microbiome with allergic condition is different from that of the gut microbiome in AC children. The microbiome profiles are changed by the western environment/lifestyle and are associated with allergies in Chinese immigrant children in Australia.

## Introduction

The rising prevalence of asthma and allergies has become a global public health concern, and there are wide variations between countries.^1^, ^2^ The prevalence of adult asthma is highest in developed countries, such as Australia (21.0%), while it is the lowest in developing countries, such as China (0.2%).^3^ The substantial difference in allergy prevalence indicates that environmental factors play a vital role in the development of these conditions.^4^ Immigrant populations in industrialized countries represent a unique opportunity to examine western environmental influences.^5^ Immigrants moving from less affluent countries (asthma-low risk) to more affluent countries (asthma-high risk) experience a gradually increased prevalence of allergies and asthma, correlated with the length of residence in the more affluent country.^6^ For example, a cross-sectional survey of school-age children reported that compared to a residence in Australia from zero to 4 years, residence for 5 to 9 years after migration was associated with a two-fold increase in reported wheezing, and this increased to a three-and-a-half-fold for 10–14 years after migration.^7^ This time-dependent effect points to a gradual change of individual homeostasis, potentially related to ongoing modifications of the human microbiome due to western environmental risk factors.

Studies have shown that perturbations in the human microbiome are associated with an increased risk of allergic disease.^8^, ^9^ This agrees with the well-known “hygiene hypothesis” that suggests early exposure of children to high microbial abundance and increased biodiversity protects against development of allergic diseases.^10^, ^11^ Our recent studies showed that Chinese immigrants in Australia had a significant shift in the innate and adaptive immune response.^12^, ^13^ Chinese immigrants living in Australia for more than 5 years had reduced innate immune cytokine production and weaker adaptive antibody responses to pathogen-associated antigens relative to recently-arrived Chinese immigrants.^12^, ^13^ We presume that the human microbiome inherent to the western environment may regulate the priming of immune response and modulate the susceptibility to allergic disorders.^14^, ^15^, ^16^ However, there is a lack of knowledge about the difference in human microbiome between immigrants and their counterparts in the country of origin. Chinese immigrant children in Australia with matched Chinese children in China are a relatively homogeneous population, yet living in an industrialized or non-industrialized environment. Therefore we compared the oropharyngeal (OP) and gut microbiome of Australian Chinese (AC) children in Australia and China-born Chinese (CC) children in mainland China. The two cohorts were strictly matched for age-range, gender-frequency, and season of recruitment to control for potential confounders.

## Methods

### Study design and recruitment

This study is a cross-sectional investigation in which the participants are living in Australia and China. First we recruited AC children from the local Chinese community living in Perth, Western Australia by advertisements through Chinese media such as radio and newspaper (from March to May 2015). Chinese children aged 3 to 18 and residing in Australia were recruited. Second we recruited CC children from cluster randomly selected students from kindergartens, primary and high schools in Hebi City in the northern Henan Province. Gender frequency and age range were matched with the AC children (from September to October 2015). The recruitment took place during autumn taking into account the countries are in opposite hemispheres. Hebi city is a relatively less affluent (prefecture-level) city in China, where agriculture has traditionally been a pillar of its economy. In total we recruited 58 AC children (aged 3–18) and 63 CC children (aged 2–17), all of whom were of Han Chinese descent.

OP swabs and fecal samples were collected from the participants and one of parents/guardians was asked to fill out a questionnaire for their child. The questionnaire collected demographic information, delivery method (Vaginal delivery/Caesarean section), breastfeeding history, self-reported food allergic history and current wheezing status. At recruitment, skin prick tests (SPT) were performed to measure the child's atopic status. The SPT results were evaluated after 15–20 ​min exposure, and positive atopy was defined as a wheal size >3 ​mm diameter in reaction to at least at one allergen (details in Supplemental Notes).^17^

This study was approved by the Human Research Ethics Committee (HREC) at the University of Western Australia. All parents provided informed consent on behalf of their children.

### 16S rRNA gene sequencing, bioinformatics and statistical analysis

Amplicons of the 16S rRNA gene V3–V4 region were sequenced on an Illumina HiSeq 2500 platform. The paired-end reads were merged, then filtered, and the sequences were assigned into Operational Taxonomic Units (OTUs) against the SILVA reference database (128 release). Bioinformatics and statistical analysis were carried out within the Quantitative Insights Into Microbial Ecology (QIIME 1.9.1) pipeline or using RStudio (Version 1.0.153). Alpha diversity, which describes the number of taxa in sites or habitats at a more local scale, was estimated using the chao1 richness estimate and Shannon index. Beta diversity, which indicates the extent of similarity between microbial communities, was measured using weighted and unweighted UniFrac. To infer the microbiome phenotypes and functional pathways associated with the bacterial taxa, we used Bugbase and Phylogenetic Investigation of Communities by Reconstruction of Unobserved States (PICRUSt) analysis. The predictions of functional pathways were collapsed into Kyoto Encyclopedia of Genes and Genomes (KEGG) Orthology groups and shown by linear discriminant analysis effect size (LEfSe) plots.

Mann-Whitney U tests were used to compare the group difference between AC and CC children, and we selected taxa with relative abundance over 1.0% to illustrate with figures and tables. Linear regression was used to investigate the associations of bacterial relative abundance and AC/CC groups, after adjusting for age, gender, BMI, breastfeeding percentage, and antibiotic usage. If the taxa that show a change in abundance in AC children relative to CC children play a role in the occurrence of allergic conditions, we expect that these taxa will show a similar change in children with allergic conditions compared to children without allergic conditions. To examine such trend, we selected taxa with significant differences (at two significance levels: p ​< ​0.05 and p ​< ​0.01) in relative abundance between AC and CC children at 5 taxonomic levels (phylum, class, order, family, and genus). Subsequently the mean difference of taxa abundance between children with positive and negative allergic conditions (atopy, food allergy, and wheezing) was calculated in AC children, and those with mean difference over 0.01% and 0.1% were selected for further analysis respectively. If the mean relative abundance of a bacterium is higher or lower in AC children (compared to CC children) and also higher or lower in children with allergic conditions (compared to children without allergic conditions), a “1” was assigned to the bacterium, otherwise a “0” was assigned. A new variable with these binomial values (1 and 0) was created for all the selected taxa and used to test for consistency using a binomial probability test. The null hypothesis for the binomial probability tests is that the proportion of 0 (inconsistent) or 1 (consistent) is equal to 50% which indicates that there is no consistent trend. We used this consistency test of the major distinct taxa to infer the influence of the western environment on the human microbiome and its relation with allergic conditions. The full methods and related references are available in Supplemental Notes.

## Results

### Characteristics of the study population

As shown in Table 1, there were no significant differences in gender, age, body mass index (BMI), delivery method, breastfed percentage and antibiotic usage between the AC and CC children. Forty-two (72.4%) of the AC children were born in Australia, and 16 (27.6%) were born in China and had been living in Australia with a median duration of 4.6 years. The percentages of atopy, food allergy, and current wheeze were all significantly higher among AC children than among CC children.

**Table 1:** The characteristics of participants.

Characteristic	AC (n ​= ​58)	CC (n ​= ​63)	*p*
**General Information**			
Females: n (%)	26 (44.8%)	30 (47.6%)	0.758
Age (y): mean (SD)	8.6 (3.5)	7.7 (3.7)	0.196
BMI (kg/m^2^): mean (SD)	17.1 (2.6)	17.6 (4.3)	0.391
Delivery method			
Vaginal delivery n (%)	34 (58.6%)	32 (50.8%)	0.701
Caesarean section n (%)	22 (37.9%)	24 (38.1%)	
Breastfed: n (%)	46 (79.3%)	53 (84.1%)	0.584
**Clinical Information**			
Antibiotic used (past 2 weeks): n (%)	2 (3.4%)	8 (12.7%)	0.179
Atopy: n (%)	36 (62.1%)	8 (12.7%)	**0.000**
Food allergy: n (%)	15 (25.9%)	5 (7.9%)	**0.017**
Wheezing: n (%)	16 (27.6%)	2 (3.2%)	**0.000**

### Microbial diversity and composition

The microbiome composition between the AC children born in China or Australia was similar and therefore grouped for further analysis. Two AC and 8 CC participants had used antibiotics 2 weeks prior to sample collection, and we performed a sensitivity test without those subjects which gave a consistent result.

### Microbial diversity

The Chao1 richness estimates are consistently lower in AC children for both OP (257.70 ​± ​43.22) and fecal samples (330.24 ​± ​41.65) compared to CC children (288.62 ​± ​43.03, 345.00 ​± ​33.39, *p* ​= ​0.002, 0.046) (Fig. 1 a and d). Conversely, the Shannon indices were significantly higher in AC children (OP: 4.46 ​± ​0.59, fecal: 5.58 ​± ​0.73) than in CC children (OP: 3.99 ​± ​0.95, fecal: 5.06 ​± ​0.67, *p* ​= ​0.009, 0.002).

**Fig. 1:**
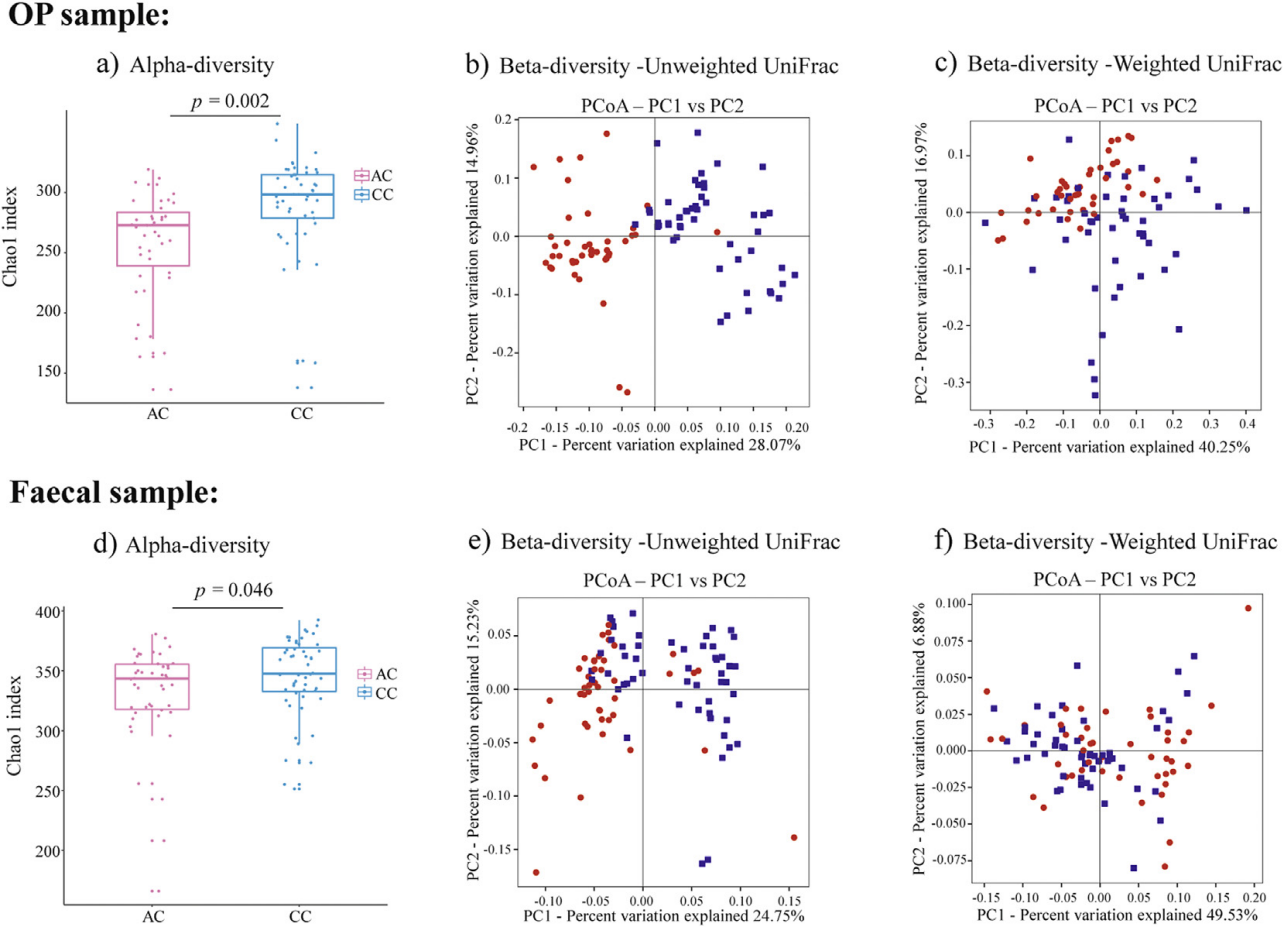
**– The alpha and beta diversity of OP and fecal samples from AC and CC children:** Alpha diversities are exemplified by the Chao1 index for oropharyngeal (OP) samples in panel **a)**, as well as fecal samples in panel **d)**. The top and bottom lines of box plots showed the interquartile range, and lines inside the boxes represented medians. Beta diversities for OP samples are represented by Principal coordinate analysis (PCoA) plots of **b)**, unweighted and **c)**, weighted UniFrac matrix, whereas those for fecal samples are similarly represented in panels **e)**, unweighted and **f)**, weighted UniFrac matrix. For beta diversity analyses, data points represent either AC samples (red) or CC samples (blue), and the 2 major principle components are respectively represented on the x- and y-axes.

A distinct clustering was observed of the OP and fecal bacteria communities between the AC and CC children using both the unweighted and weighted UniFrac matrix presented by Principal Coordinate Analysis (PCoA) plots (Fig. 1 b, c, e, f). Additionally, ANOSIM and Adonis statistical model analyses further showed a significant difference between both OP and fecal bacterial communities of AC and CC children (Supplemental Table1).

### Oropharyngeal sample bacterial composition

A total of 16 bacterial phyla were detected from OP swabs (Fig. 2a, Supplemental Table2). Phylum-level taxonomical assignment showed that *Firmicutes* and *Proteobacteria* were dominant in both AC (49.6%, 19.8%) and CC children (46.2%, 26.6%). At the genus level, 12 genera accounted for 80.8% of the abundance in AC, and 15 genera accounted for 82.3% of the abundance in CC children, using a minimum relative abundance of 1.0% (Supplemental Figure 1a, Supplemental Table2).

**Fig. 2:**
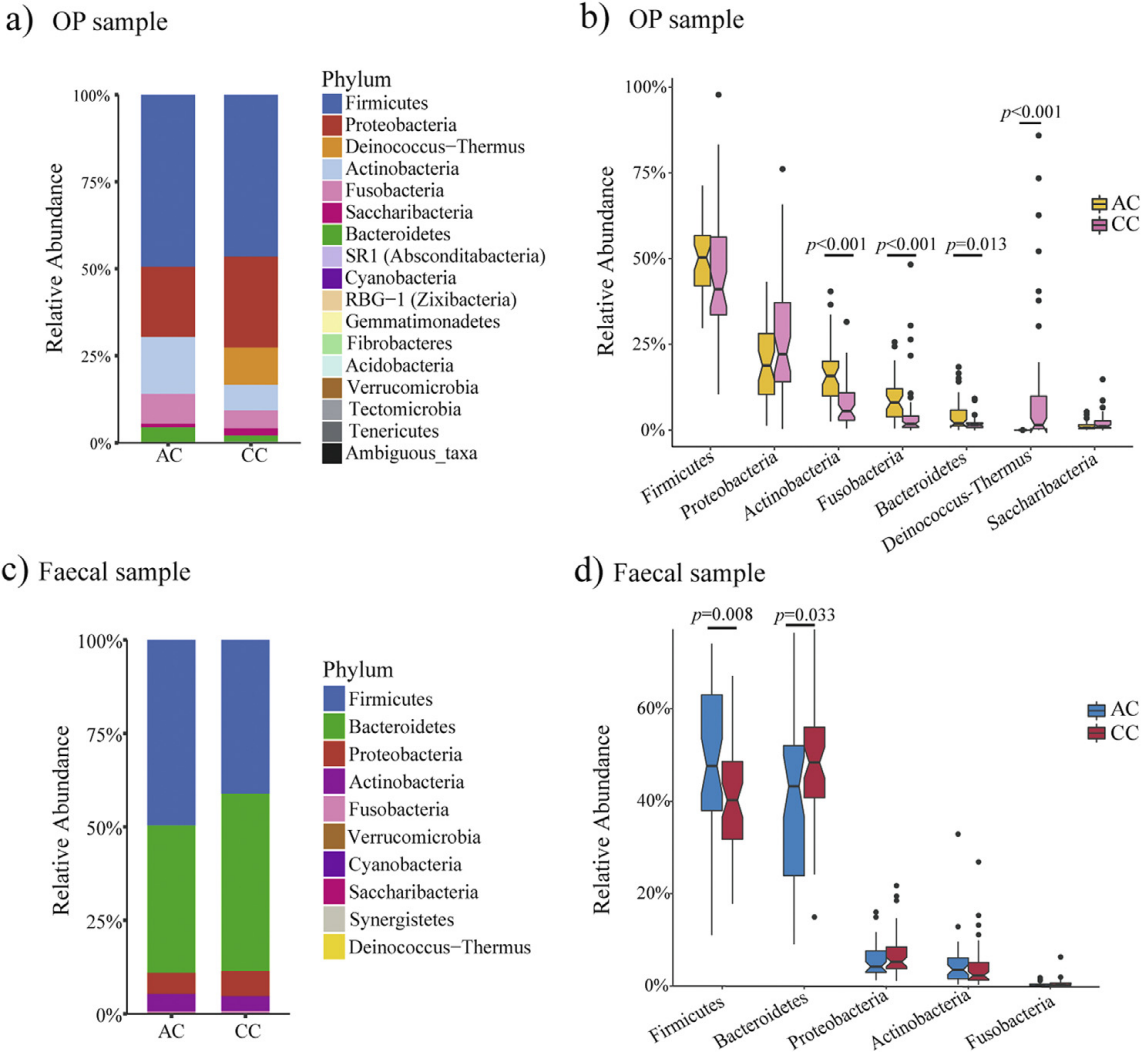
**– Bacteria relative abundance composition and comparison of oropharyngeal and fecal samples at the phylum level:** The composition of bacterial relative abundance are shown in bar plots: panel **a)** oropharyngeal (OP) samples and **c)** fecal samples. The major phyla (relative abundance >1.0%) comparisons between AC and CC children are shown on box plots in panel **b)** OP samples and **d)** fecal samples. The top and bottom lines of box plots show the interquartile range, and lines inside the boxes represent medians, and black dots represent outliers.

Among the total of 16 bacterial phyla and 193 genera, 6 (37.5%) phyla and 113 (58.5%) genera were significantly different between AC and CC children. AC children had a lower proportion of phylum Deinococcus-Thermus (*p* ​< ​0.001), and higher proportions of Actinobacteria (*p* ​< ​0.001), Fusobacteria (p ​< ​0.001), and Bacteroidetes (*p* ​= ​0.013) (Fig. 2b, Supplemental Table2). The differences between the two groups of children are shown for the 10 most abundant genera in Supplemental Figure 1b. These differences remained significant after further adjustment for confounders (age, gender, BMI, breastfed percentage, and antibiotic use) using linear regression.

### Fecal sample bacterial composition

We observed 10 distinct phyla in the fecal microbiomes of AC and CC children (Fig. 2c, Supplemental Table3). The phylum Firmicutes (49.4%) was dominant in AC children, whereas the phylum Bacteroidetes (47.5%) had the highest proportion in CC children. These 2 phyla made up the vast majority of OTUs, namely 89.3% in AC and 88.4% in CC children. Seven (70.0%) phyla and 91 (62.8%, 91/145) genera were significantly different between AC and CC children, with the major differences shown in Fig. 2d, Supplemental Figure 2. After adjusting for confounders, the relative abundance of phyla Firmicutes, and Bacteroidetes, genera *Ruminococcus1*, *Lachnospira*, *Eubacterium*, *Peptoclostridium*, *Barnesiella*, *Parasutterella*, and *Escherichia-Shigella* remained significantly different between AC and CC children.

### Trend consistency of taxonomic abundance with the western environment and allergy

We identified taxa that were different between the AC and CC children across the 5 taxonomic levels. We selected 204 OP taxa with a significance level *p* ​< ​0.05 and 141 OP taxa with a significance level *p* ​< ​0.01. For fecal samples this was 123 and 81 taxa, respectively. Combined with the mean difference of taxa abundance between positive and negative allergic conditions (>0.01% and >0.1%) we performed 4 sets of binomial tests each for atopy, food allergy, and wheezing among AC children (Supplemental Table5).

We discuss the binomial test results for the analysis of *p* ​< ​0.05 and difference >0.01% (Supplemental Table5). In OP samples, 84.0% (63/75) of the taxa showed a consistent trend for food allergy that is significantly higher than 50% (*p* ​< ​0.001). Such trend was also found for the fecal taxa and atopy (61.0% (36/59)). In contrast, only 23.6% (13/55) and 29.8% (14/47) of the fecal taxa showed a consistent trend for food allergy and wheezing, significantly lower than 50% with a *p* value of <0.001 and 0.008, respectively. This indicates that the trend is inverse, an increase in abundance of the fecal taxa in AC children corresponds to a decrease in these taxa in children with food allergy or wheezing. These findings are observed for all the 4 analyses presented in Supplemental Table5 albeit different cut-off points of significance and difference. Fig. 3 shows the proportion of consistency in OP and fecal samples for food allergy and wheezing.

**Fig. 3:**
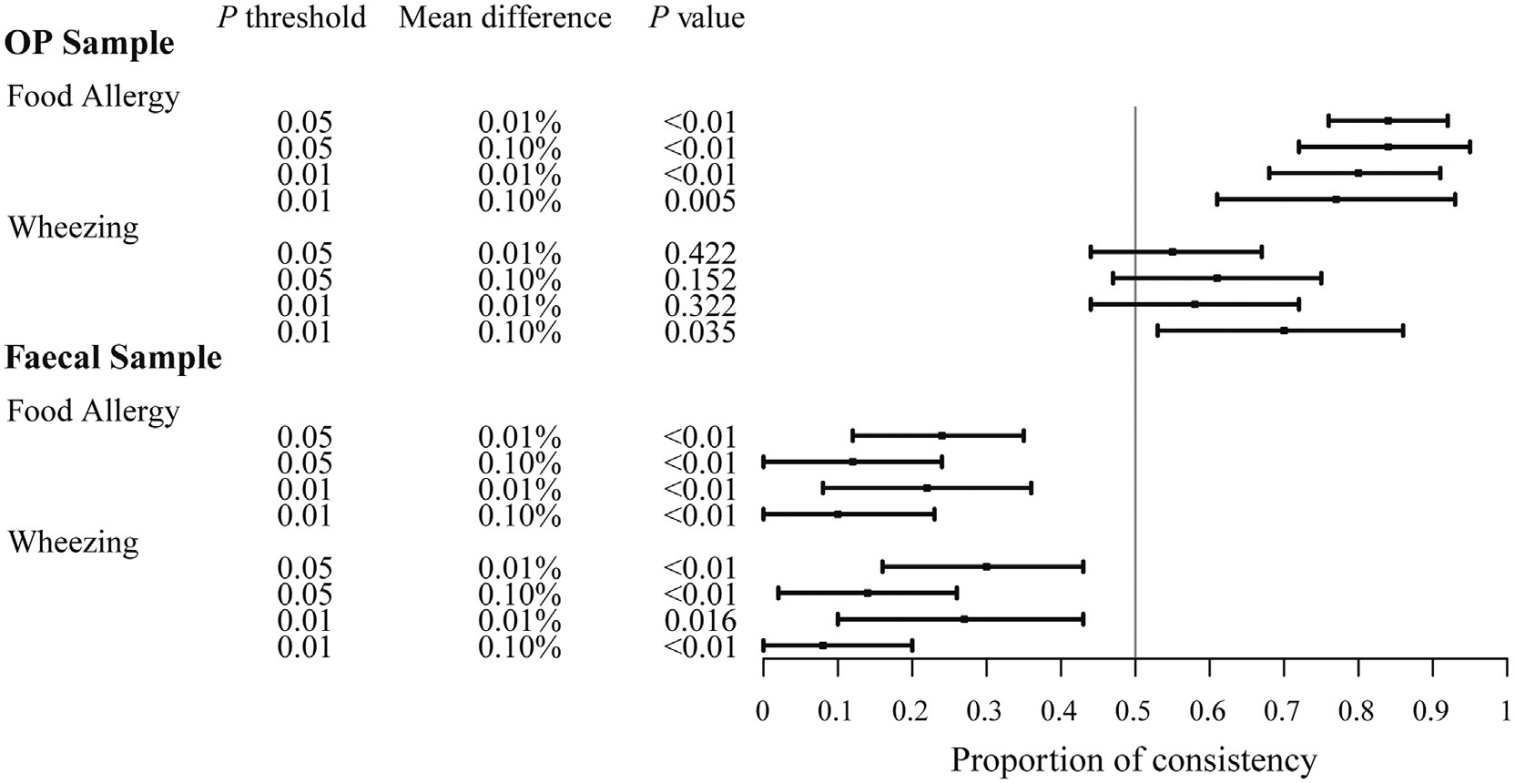
**– Consistency in trends of taxa abundance between the oropharyngeal (OP) and fecal microbiome:** Left columns indicate the taxa selection thresholds *p* values (0.05 and 0.01) for the abundance comparison between AC and CC children, and the mean difference between with or without allergic conditions (over 0.01% or 0.10%). The “P value” column represents the significance of each binomial probability test. The horizontal axis of the plot represents the proportion of consistency of taxa abundance that is higher or lower in AC children (compared to CC children) and in children with allergic conditions (relative to children without allergies) among AC children. At the vertical “line of null effect” there is no consistent trend. Each horizontal line on the plot represents a consistent trend under certain thresholds. The black box indicates the mean value of the proportion of consistency, and the horizontal line represents the 95% confidence intervals.

## Discussion

This is the first study that compares the diversity and composition of human OP and fecal microbiomes in a single ethnic (Han) group of children living in either a Western (Australia) or Eastern (China) environment. Here, we use the term *western environment* as a phrase to collectively denote the socio-cultural, lifestyle, and geographical environment in industrialized countries, such as Australia, and the *eastern environment* as a collective term to indicate non-industrialized counties, such as China. The children were matched in age and gender, and we also matched the recruitment season in Australia and China. We selected this homogenous population to control the genetic influence so that only environmental exposure is varied, which is a main advantage of this study.

As expected, AC children had higher rates of atopy, food allergy, and wheezing compared to CC children. We hypothesized that the Australian environment has modified the human microbiome in Chinese immigrant children (microbiome modification by the western environment), thereby leading to more allergy in AC children (the western microbiome causing allergy). Consistent with the first part of our hypothesis, we found significant differences in microbial diversity, composition, and functional pathway expression in both OP and fecal microbiota between Chinese children living in Australia and China. We designed the study to measure significant differences in the microbiome between AC and CC children, rather than identifying specific taxa associated with allergic conditions in the population. To examine the hypothesis of the western microbiome relating to allergy we investigated if the change in relative abundance of taxa in AC children (compared to CC children) is also apparent in children with allergic conditions (relative to children without allergies) among the AC population to examine the hypothesis of the western microbiome relating to allergy. Such a consistent trend was significant (>50%) for food allergy and wheezing in the OP microbiome. This means that OP taxa which increase/decrease in the western environment are likely to show the same increase/decrease in children with food allergy and wheezing. In contrast, we found that such trend was significantly lower than 50% for fecal samples with these two phenotypes. fecal taxa which increase/decrease in the western environment likely show an opposite effect, namely decrease/increase, in children with food allergy or wheezing. Conceivably, the environment of upper respiratory tract and gastrointestinal tract are very different, and the way the microbial component interacts with the immune system differs considerably. The gut has numerous immunogenic regions, where microbial elements interact rapidly with regulatory T cells.^18^ The segmented filamentous bacteria have the function of promoting intestinal T helper type (Th17) responses.^19^ Therefore, the mechanism of interaction could be very different between the two sites as is the diversity and taxonomic groups in those two distinct areas.

This is a cross-sectional study and these trend consistencies do not indicate a cause-effect relationship. Children with food allergy and wheezing may have a changed immune status that changes the abundance of taxa. This may partly explain the inverse trend mentioned above. We think that the observed trends are unlikely to be false findings as they are consistently significant in all 4 analyses. Our study shows that western oropharyngeal and gut microbial flora are associated with allergic conditions in Chinese immigrant children. More studies are required to clarify the opposite trend that is observed for OP and gut microbiomes with food allergy and wheezing in industrialized countries.

Environmental biodiversity is important for human health. Lynch et al. reported that healthy children were exposed to richer and more diverse bacterial communities in the first year of life, compared to those children that developed either atopy or recurrent wheeze.^20^ Another recent study compares the prevalence of asthma between Amish and Hutterites schoolchildren (similar genetic ancestries and lifestyle). It revealed that Amish children, living on a traditional farm, have been exposed to a more enriched microbiota environment and demonstrate low rate of asthma, compared to Hutterites children whose farming practice is industrialized.^21^ There is emerging evidence that the environmental influence (environmental microbiomes) on shaping human microbiomes is a key element in tuning immune system and development of allergy. Several studies have shown that a reduced diversity of the human microbiome may be a risk for asthma and allergy.^22^ A low microbial diversity in early infancy can potentially predict atopic dermatitis.^23^ A longitudinal study demonstrated a lower oral bacterial diversity among children who developed allergic disease, particularly asthma at an age of 7 years.^24^ There are different indexes that estimate microbial diversity such as the Chao1 index as a richness estimator and the Shannon index for the bacterial evenness.^25^ In our study both OP and fecal samples in AC children had a lower Chao1 index but a higher Shannon index. This indicates that the western environment has shaped the microbiome to have less richness and more evenness. In another population comparison study it was found that the alpha-diversity of the fecal microbiome (Chao1 and Shannon indexes) was higher in African children (non-industrialized) compared to those of European (industrialized environment like Australia) children.^26^ The findings of these studies are largely consistent with our study. Microbial diversity variations in the human microbiome related to the western environment may provide a mechanistic explanation for the allergy epidemic in the past 60 or 70 years.

The microbiome profiles in AC children are significantly different from CC children. This indicates that western and eastern populations may be living with a different genus and species group of commensal microorganisms. This present study is not designed to ascertain which bacteria that are commonly present in western populations cause allergy, as it is likely a combination effect of many. Rather we analyze the difference between microbiome profiles in western and eastern populations to aid further studies to clarify their causal effects on asthma and allergy.

We discuss a few dominant taxa and compare our findings with recent literature. The genus *Streptococcus* (Firmicutes), a Gram-positive bacterium, has the largest abundance in both AC and CC children in OP samples but in a significantly higher proportion among AC children compared to CC children. Studies have shown that *Streptococcus* is associated with allergic symptoms. A 234 children cohort study revealed that early colonization of *Streptococcus* in the nasopharyngeal microbiota was a strong predictor for asthma during the first year of life, and its colonization was linked to atopy by the age of two years and chronic wheeze at age five.^27^ Similarly, another study of neonatal oropharynx bacteria showed that a high burden of *Streptococcus* within the first month of life increased the risk for recurrent wheeze and asthma development.^28^ That the gut microbiota is critical for immune development has been well documented.^29^ The majority of genera that showed a significantly higher abundance in AC children compared to CC children were in the class Clostridia of phylum Firmicutes. Interestingly, a recent study found that the same class and phylum were enriched in fecal microbiome of food-allergic children compared to siblings and healthy children, but other *Clostridium* species were enriched in non-food-allergic subjects.^30^ Class Clostridia has been associated with immune tolerance in mouse models of allergy and aids protection from allergic inflammation.^31^ Our inverse association between the western fecal microbiome and food allergy and wheezing partly supports the association of the class Clostridia with food allergy reported in the literature.

The cell walls of Gram-negative bacteria contain lipopolysaccharide (LPS), which contribute to innate immune tolerance and help to prevent inappropriate immune stimulation through the microbiota-epithelial crosstalk.^32^ Indeed, we found Gram-negative bacteria were higher among CC children in both OP and fecal samples using BugBase. Although the KEGG pathways provide limited understanding of the actual bacterial potential functions, differences in the expression of certain pathways can indicate potential associations.

One limitation is that this study is cross-sectional, and the results cannot determine causality. To Chinese migrants, the change to a western environment is the combination of a different diet, less air pollution, exposure to new allergens and, greater hygiene, all of which can lead to different microbiota composition/diversity, and contribute to the increased allergies in AC children.^33^, ^34^ However, in this study we could only focus on the overall influence of a western environment on the microbiome and the relation with allergy. Thirdly, recent antibiotic usages are known to have a significant impact on the human microbiome. Antibiotic use during the two-week period prior to the recruitment was 3 to 4 times more common in CC than in AC children. The disparity of antibiotic use in the two population may confound the findings in this study. Unfortunately, we did not collect a detailed history of antibiotic use in this population. In addition, the resolution of 16s rRNA sequencing is reliable down to the genus level. Studies utilizing whole-genome sequencing or real-time PCR, are of interest to further investigate the species and strains of bacteria that are different between the industrialized and non-industrialized environment, as well as to understand how the western microbiome shapes the immune system, leading to the development of asthma and allergy. Moreover, a comparison of microbiota and allergies present before and after immigration is worthy of investigating for future study.

## Conclusion

We found evident differences in the compositions of the OP and gut microbiome between AC and CC children. The AC children demonstrated a lower microbial diversity richness and higher diversity evenness compared to CC children. The association of the OP microbiome with food allergy and wheezing is different from the gut microbiome in Chinese immigrant children in Australia. The western environment/lifestyle promotes a different human microbiome profile that may significantly contribute to the increased prevalence of asthma and allergy in industrialized countries.

## Competing interests

The authors declare that they have no competing interests.

## Ethics approval and consent to participate

This study was approved by the Human Research Ethics Committee (HREC) at the University of Western Australia. All parents provided an informed consent on behalf of their child.

## Authors' contributions

J.G. was responsible for the data analysis, interpretation, and writing and revising the manuscript. Q.L., X.W., and A.S. contributed to the study design, recruitment, and data acquisition. A.A., X.Z., C.S.P, Y.S., P.E.M., G.A.D., and E.K.M verified the analytical methods, and contributed to manuscript revision. G.Z., F.L., and P.N.L. designed and directed the study, provided critical revision of the manuscript. G.Z. supervised all aspects of the study. All authors read and approved the final manuscript.

## Funding

The study was funded by Telethon Perth Children's Hospital Research Funds.

## Figures and Tables

**Figs1:**
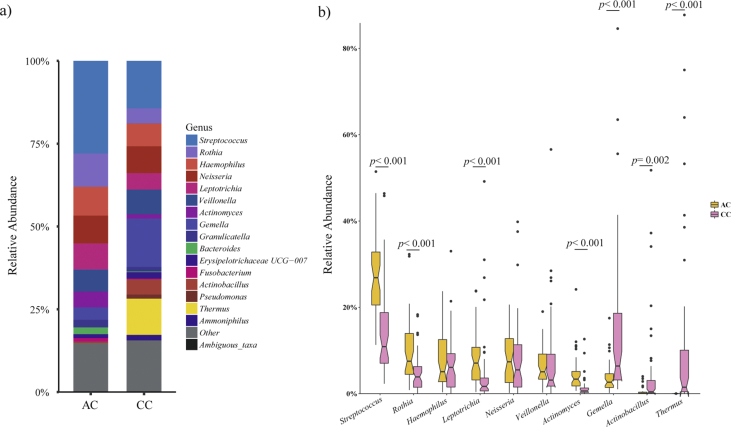
**Supplementary Figure 1- Bacterial relative abundance composition and comparison of oropharyngeal samples at the genus level: a)** The bar plot shows the composition of bacterial relative abundance. Major genera (relative abundance >1.0%) are drawn individually and taxa with relative abundances less than 1.0% are combined into the ‘Other’ category. **b)** The box plot shows the comparison of top ten major genera in oropharyngeal samples between AC and CC children. The notched boxes show the interquartile range, and lines inside the boxes represent medians, and black dots represent outliers.

**Figs2:**
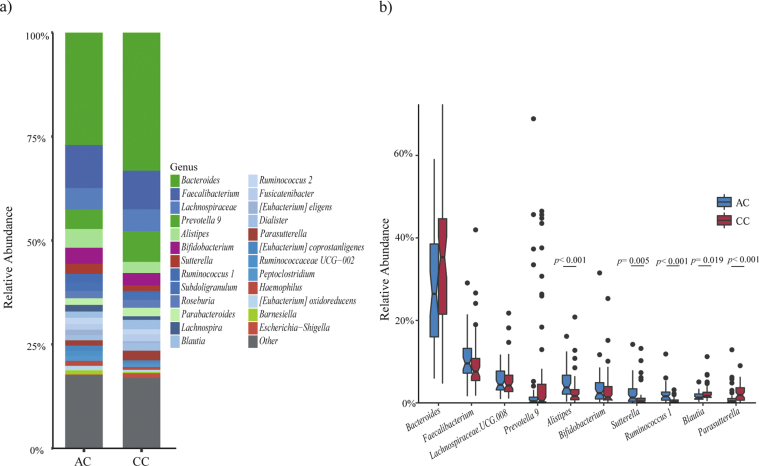
**Supplementary Figure 2- Bacterial relative abundance composition and comparison of faecal samples at the genus level: a)** The bar plot shows the composition of bacterial relative abundance. Major genera (relative abundance >1.0%) are drawn individually and taxa with relative abundances less than 1.0% are combined into the ‘Other’ category. **b)** The box plot shows the comparison of top ten major genera in faecal samples between AC and CC children. The notched boxes show the interquartile range, and lines inside the boxes represent medians, and black dots represent outliers.

**Figs3:**
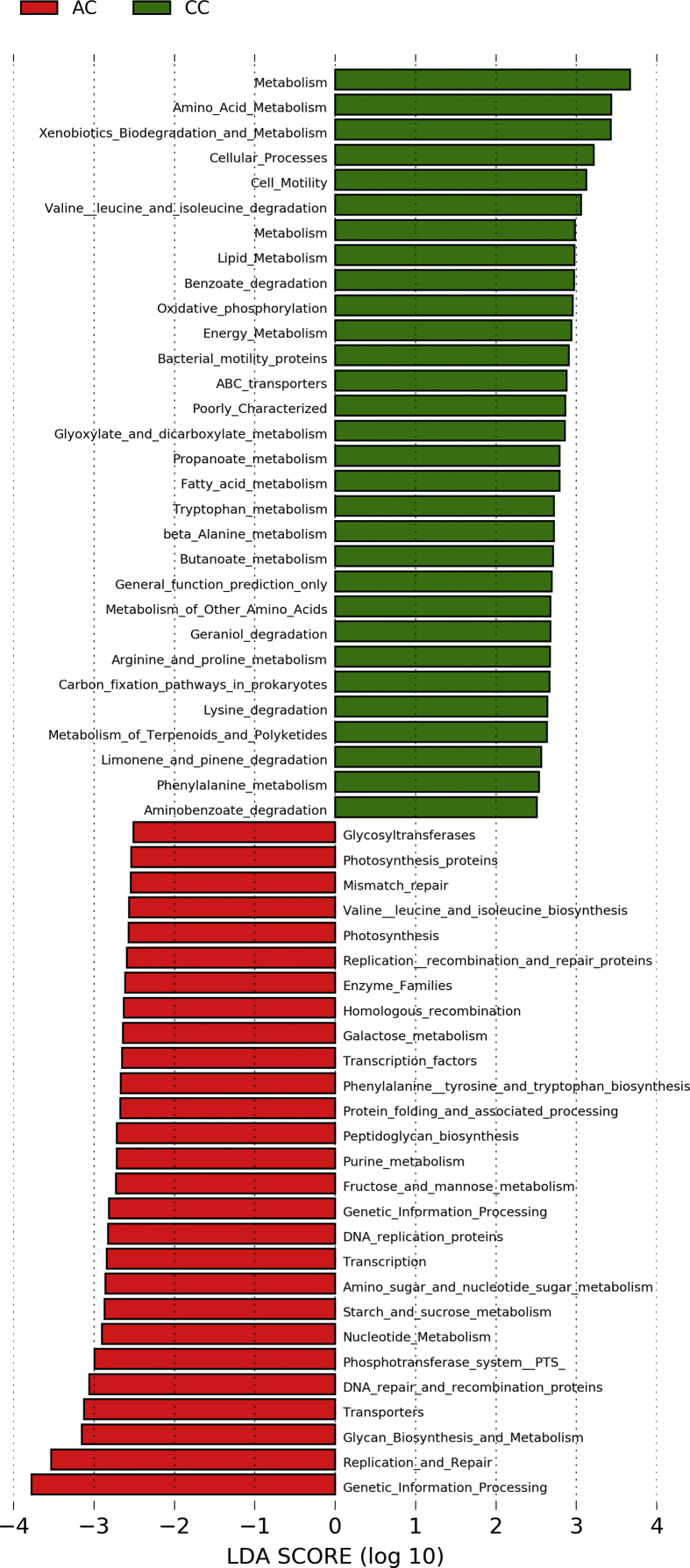
**Supplementary Figure 3 –Bar graphs of linear discrimination analysis (LDA) for the mean differences of KEGG pathway for oropharyngeal samples:** KEGG pathway more present in Australian Chinese (AC) children are represented by red bars, whereas those more abundant in China-Born Chinese (CC) children are represented by green bars. Note the plots are a logarithmic x-axis (of base 10) and only KEGG pathway difference for an order of 2.0 or higher are represented.

**Figs4:**
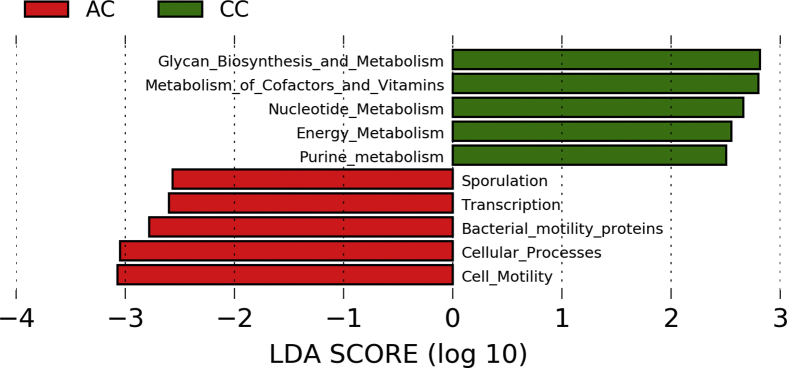
**Supplementary Figure 4 – Bar graphs of linear discrimination analysis (LDA) for the mean differences of KEGG pathway for faecal samples:** KEGG pathway more present in Australian Chinese (AC) children are represented by red bars, whereas those more abundant in China-Born Chinese (CC) children are represented by green bars. Note the plots are a logarithmic x-axis (of base 10) and only KEGG pathway difference for an order of 2.0 or higher are represented.
