# Supplementary material for: Western oropharyngeal and gut microbial profiles are associated with allergic conditions in Chinese immigrant children
Source: World Allergy Organ J. 2019 Aug 9;12(8):100051. doi: 10.1016/j.waojou.2019.100051 (PMC6699559; doi:10.1016/j.waojou.2019.100051)
Supplement: Multimedia component 1 [file mmc1.doc]

**Online Supplemental Notes**

**Description of methods:**

**Skin Prick Test (SPT)**

The skin prick tests (SPT) were performed on every child by experienced nurses in order to measure atopic status with commercial allergen extracts kits. In Australia, the common allergens include cow’s milk, egg white, rye grass, mixed grass, Dermatophagoides farinae, Dermatophagoides pteronyssinus, cat dander, dog dander, cockroach and moulds. In China, the common allergens were the same as those in Australia, except for added ragweed and shrimp, and omitted rye grass and mixed grass.

**Sample collection and processing**

OP swabs were collected with a sterile cotton swab and a collection tube and were immediately placed on dry ice and transported to the laboratory freezer (-80°C). Parents were asked to collect faecal samples from their child in a 25 ml faeces container (SARSTEDT) provided by our laboratory. They collected approximately 10 grams of voided stool that was immediately frozen at −20°C in a freezer at home. The samples were then collected by research members within 3 days and transported on dry ice to the laboratory freezers (-80°C) for storage until analysis.

In total, 45 OP samples and 46 faecal samples were collected from participants in Australia. Sixty two OP samples and 53 faecal samples were collected from participants in China. 13 OP samples and 1 faecal samples from China did not pass the DNA quality control. The rest of the samples were selected for further analysis. DNA from OP and faecal samples was extracted using the QIAamp DNA Microbiome Kit (cat#51704, QIAGEN) following the instructions from the manufacturer. The DNA concentration was monitored by Nanodrop (ND-1000, Thermo scientific).

**16S rRNA gene sequencing**

The bacterial 16s rRNA gene V3-V4 regions were amplified by using primers 341F (5’-CCTAYGGGRBGCASCAG-3’) and 806R (5’-GGACTACNNGGGTATCTAAT-3’) with the barcode. PCR reaction was carried out with Phusion® High-Fidelity PCR Master Mix (New England Biolabs). The cycling conditions were: 98℃ for 1 min, 35 cycles of 98℃ for 10 sec, 50℃ for 30 sec, 72℃ for 30 sec; and then final extension at 72℃ for 5 min.

The resulting amplicons were mixed with the same volume of 1X loading buffer. After operating electrophoresis on 2% agarose gel, samples with a bright main strip between 400-450bp were chosen for further experiments. PCR products were mixed in equidensity ratios. Then, mixture PCR products were purified using QIAGEN Gel Extraction Kit (QIAGEN, Germany).

Sequencing libraries were generated using TruSeq® DNA PCR-Free Sample Preparation Kit (Illumina, USA). The library quality was assessed on the Qubit@ 2.0 Fluorometer (Thermo Scientific) and Agilent Bioanalyzer 2100 system. At last, the library was sequenced on an Illumina HiSeq 2500 platform. We generated 4,166,867 and 4,606,736 sequence reads that passed the quality check from 16S rRNA gene V3-V4 region of Oropharyngeal (OP) and faecal samples, respectively.

**Bioinformatics analysis**

Paired-end reads were merged using FLASH V1.2.7 [1]. Raw sequencing reads were filtrated by using Quantitative Insights Into Microbial Ecology (QIIME 1.9.1) (http://qiime.org/scripts/split_libraries_fastq.html) according to: (1) read-quality score not less than 19; (2) setting length not to fall below 3bp; (3) consecutive high quality base over 75%. Chimera sequences were removed with the usearch61 algorithm (<http://qiime.org/scripts/identify_chimeric_seqs.html>) [2]. Sequences were assigned into Operational Taxonomic Units (OTUs) by using the open reference OTU picking workflow against the SILVA reference database (128 release) [3] at ≥ 97% sequence identity threshold (http://qiime.org/scripts/pick_open_reference_otus.html). The total number of sequences were discard by filtering the observation counts below 0.005% [4]. OTUs were single rarefied to get even depths of 19785.0 for OP and 27240.0 for faecal samples, respectively. The α- diversity, which describes the number of taxa in sites or habitats at a more local scale, was determined by using the statistical methods: chao1 richness estimate [5], and Shannon index [6] by using a nonparametric two sample t-test with 999 of Monte Carlo permutations (http://qiime.org/scripts/compare_alpha_diversity.html). The β-diversity analysis indicates the extent of similarity between microbial communities [7]. The distances/similarities between microbial communities were calculated using weighted and unweighted UniFrac matrix.

To predict the biologically interpretable microbiome phenotypes as well as functional pathways of the microbiome, we clustered the sequences into OTUs against the Greengenes reference database (13_8 release) to use Bugbase [8], and to perform Phylogenetic Investigation of Communities by Reconstruction of Unobserved States (PICRUSt) analysis [9]. The estimated abundances from the Kyoto Encyclopedia of Genes and Genomes (KEGG) Orthology groups [10] were compared between AC and CC children using the linear discriminant analysis effect size (LEfSe) with the linear discriminant analysis (LDA) cutoff of 2.5 [11].

**Statistical analysis**

Statistical analysis was carried out using RStudio (Version 1.0.153) or within the QIIME pipeline. Mann-Whitney U tests were used to find different OTU relative abundance between 2 groups by using “group_significance.py” within QIIME. Alpha-diversity metrics were performed by “compare_alpha_diversity.py”, and ANOSIM and Adonis methods (“compare_categories.py”) were used to analyze beta-diversity difference in QIIME. Data were visualized by bar plots or box plots using the “ggplot2” package in RStudio. All the p-values were False Discovery Rate (FDR)-corrected to control for multiple testing.

**Results:**

**Oropharyngeal sample bacterial composition**

The relative abundance of genus *Thermus* (phylum *Deinococcus-Thermus*) was lower in AC children, whereas genera *Rothia*, *Actinomyces* (phylum *Actinobacteria*), *Leptotrichia*, *Fusobacterium* (phylum *Fusobacteria*), and *Bacteroides* (phylum *Bacteroidetes*) were all present in significantly higher proportions in AC. The *Firmicutes* did not show a difference at phylum level, however the genera *Streptococcus* and *Granulicatella* were present in higher proportions in AC children, while the genera *Gemella*, *Ammoniphilus* were present in higher proportions in CC children. In addition, the genera of the phylum *Proteobacteria*, including *Actinobacillus*, *Pseudomonas*, and *Sphingomonas* were all present in higher proportions in CC children.

**Faecal sample bacterial composition**

The phylum Firmicutes was more abundant in AC children (*p*= 0.008), and included the genera *Ruminococcus1*, *Lachnospira*, *Ruminococcaceae UCG-002*, *Eubacterium*, and *Peptoclostridium*. The genus *Blautia* was present in a significantly higher frequency in CC children (*p*= 0.019)*.* The phylum Bacteroidetes was more abundant in CC children (*p*= 0.033), but the downstream genera *Alistipes* and *Barnesiella* were significantly higher in AC children (*p*= 0.001, and *p*= 0.001). Within the phylum Proteobacteria, the genus *Sutterella* was present in a significantly higher frequency in AC children, while *Parasutterella*, and *Escherichia-Shigella* were more abundant in CC children (*p*<0.001).

**Functional characterization of the microbiome communities**

The OP microbiome of AC children had a higher frequency of Gram-positive, anaerobic, facultative anaerobe and potentially pathogenic bacteria, while the OP microbiome of CC children had a higher frequency of Gram-negative bacteria. The Gram-reaction groupings as well as the facultative anaerobic bacteria findings were the same for the faecal samples of both groups (Supplemental Table4). The KEGG pathways showed larger disparities in functional profiles between AC and CC children in OP samples, compared to faecal samples (Supplemental Figure 3 and 4).

For OP samples comparison, genetic information processing (DNA repair and recombination proteins), environmental information processing (transporters and phosphotransferase system PTS) and glycan biosynthesis and metabolism were increased most significantly (LDA > 3) in AC children relative to CC children, whereas xenobiotics biodegradation and metabolism, amino acid metabolism (valine leucine and isoleucine degradation) and cellular process (cell motility) were increased most significantly among CC children (LDA >3, Supplemental Figure 3). For faecal samples, cellular process (cell motility) and genetic information processing (transcription) were increased in AC children, while several metabolic pathways were increased in CC children (Supplemental Figure 4).

**Reference**

1. Magoc T, Salzberg SL. FLASH: fast length adjustment of short reads to improve genome assemblies. Bioinformatics. 2011;27(21):2957-63.

2. Caporaso JG, Kuczynski J, Stombaugh J, Bittinger K, Bushman FD, Costello EK, et al. QIIME allows analysis of high-throughput community sequencing data. Nature methods. 2010;7(5):335-6.

3. Quast C, Pruesse E, Yilmaz P, Gerken J, Schweer T, Yarza P, et al. The SILVA ribosomal RNA gene database project: improved data processing and web-based tools. Nucleic Acids Res. 2013;41(Database issue):D590-6.

4. Bokulich NA, Subramanian S, Faith JJ, Gevers D, Gordon JI, Knight R, et al. Quality-filtering vastly improves diversity estimates from Illumina amplicon sequencing. Nature methods. 2013;10(1):57-9.

5. Chao A. Nonparametric-Estimation of the Number of Classes in a Population. Scand J Stat. 1984;11(4):265-70.

6. Shannon CE. The mathematical theory of communication (Reprinted). M D Comput. 1997;14(4):306-17.

7. Lozupone C, Knight R. UniFrac: a new phylogenetic method for comparing microbial communities. Appl Environ Microbiol. 2005;71(12):8228-35.

8. Ward T, Larson J, Meulemans J, Hillmann B, Lynch J, Sidiropoulos D, et al. BugBase Predicts Organism Level Microbiome Phenotypes. bioRxiv. 2017:133462.

9. Langille MGI, Zaneveld J, Caporaso JG, McDonald D, Knights D, Reyes JA, et al. Predictive functional profiling of microbial communities using 16S rRNA marker gene sequences. Nat Biotechnol. 2013;31(9):814-21.

10. Kanehisa M, Goto S. KEGG: kyoto encyclopedia of genes and genomes. Nucleic Acids Res. 2000;28(1):27-30.

11. Segata N, Izard J, Waldron L, Gevers D, Miropolsky L, Garrett WS, et al. Metagenomic biomarker discovery and explanation. Genome Biol. 2011;12(6):R60.
