# Supplementary material for: Western oropharyngeal and gut microbial profiles are associated with allergic conditions in Chinese immigrant children
Source: World Allergy Organ J. 2019 Aug 9;12(8):100051. doi: 10.1016/j.waojou.2019.100051 (PMC6699559; doi:10.1016/j.waojou.2019.100051)
Supplement: Multimedia component 2 [file mmc2.doc]

**Supplemental Tables**

**Supplemental Table 1.**

**The p-values of β-diversity comparison between AC and CC children**

| ***β*-diversity** | **OP samples** | | **faecal samples** | |
| --- | --- | --- | --- | --- |
|  | ANOSIM | Adonis | ANOSIM | Adonis |
| Unweighted UniFrac PCoA | 0.001 | 0.001 | 0.001 | 0.001 |
| Weighted UniFrac PCoA | 0.001 | 0.001 | 0.001 | 0.003 |

**Supplemental Table 2.**

**The phylum and genus level of OP samples microbiome compared** between AC and CC children

| **Phylum** | **AC** | **CC** | ***p*** | ***FDR_p**** |
| --- | --- | --- | --- | --- |
| Firmicutes | 49.63% | 46.22% | 0.186 | 0.198 |
| Proteobacteria | 19.80% | 26.57% | 0.149 | 0.181 |
| Actinobacteria | 16.04% | 7.61% | 0.000 | **0.000** |
| Fusobacteria | 8.92% | 4.95% | 0.000 | **0.000** |
| Bacteroidetes | 4.41% | 1.83% | 0.005 | **0.013** |
| Saccharibacteria | 1.13% | 2.12% | 0.045 | 0.086 |
| Cyanobacteria | 0.02% | 0.08% | 0.000 | 0.001 |
| SR1 (Absconditabacteria) | 0.02% | 0.11% | 0.046 | 0.086 |
| Tenericutes | 0.01% | 0.00% | 0.015 | 0.036 |
| Deinococcus-Thermus | 0.00% | 10.34% | 0.000 | **0.000** |
| Fibrobacteres | 0.00% | 0.01% | 0.120 | 0.157 |
| RBG-1 (Zixibacteria) | 0.00% | 0.06% | 0.101 | 0.143 |
| Gemmatimonadetes | 0.00% | 0.04% | 0.101 | 0.143 |
| Tectomicrobia | 0.00% | 0.01% | 0.101 | 0.143 |
| Acidobacteria | 0.00% | 0.01% | 0.183 | 0.198 |
| Verrucomicrobia | 0.00% | 0.01% | 0.349 | 0.349 |
| Ambiguous taxa | 0.01% | 0.04% | 0.000 | 0.000 |
| **Genus** |  |  |  |  |
| *Streptococcus*  (Firmicutes; Bacilli; Lactobacillales; Streptococcaceae) | 26.59% | 13.91% | 0.000 | **0.000** |
| *Rothia*  (Actinobacteria; Actinobacteria; Micrococcales; Micrococcaceae) | 9.47% | 4.69% | 0.000 | **0.001** |
| *Haemophilus*  (Proteobacteria; Gammaproteobacteria; Pasteurellales; Pasteurellaceae) | 8.18% | 6.65% | 0.287 | 0.344 |
| *Leptotrichia*  (Fusobacteria; Fusobacteriia; Fusobacteriales; Leptotrichiaceae) | 7.80% | 4.68% | 0.000 | **0.000** |
| *Neisseria*  (Proteobacteria; Betaproteobacteria; Neisseriales; Neisseriaceae) | 7.77% | 8.01% | 0.555 | 0.595 |
| *Veillonella*  (Firmicutes; Negativicutes; Selenomonadales; Veillonellaceae) | 6.31% | 7.41% | 0.087 | 0.128 |
| *Actinomyces*  (Actinobacteria; Actinobacteria; Actinomycetales; Actinomycetaceae) | 4.44% | 1.29% | 0.000 | **0.000** |
| *Gemella*  (Firmicutes; Bacilli; Bacillales; Family XI) | 3.60% | 13.79% | 0.000 | **0.000** |
| *Granulicatella*  (Firmicutes; Bacilli; Lactobacillales; Carnobacteriaceae) | 2.13% | 1.31% | 0.001 | **0.004** |
| *Bacteroides*  (Bacteroidetes; Bacteroidia; Bacteroidales; Bacteroidaceae) | 1.90% | 0.23% | 0.002 | **0.004** |
| *Fusobacterium*  (Fusobacteria; Fusobacteriia; Fusobacteriales; Fusobacteriaceae) | 1.12% | 0.26% | 0.000 | **0.000** |
| *Erysipelotrichaceae UCG-007*  (Firmicutes; Erysipelotrichia; Erysipelotrichales; Erysipelotrichaceae) | 1.08% | 1.85% | 0.315 | 0.368 |
| *Actinobacillus*  (Proteobacteria; Gammaproteobacteria; Pasteurellales; Pasteurellaceae) | 0.42% | 4.53% | 0.000 | **0.002** |
| *Pseudomonas*  (Proteobacteria; Gammaproteobacteria; Pseudomonadales; Pseudomonadaceae) | 0.01% | 1.23% | 0.000 | **0.000** |
| *Sphingomonas*  (Proteobacteria; Alphaproteobacteria; Sphingomonadales; Sphingomonadaceae) | 0.00% | 1.06% | 0.000 | **0.000** |
| *Thermus*  (Deinococcus-Thermus; Deinococci; Thermales; Thermaceae) | 0.00% | 10.34% | 0.000 | **0.000** |
| *Ammoniphilus*  (Firmicutes; Bacilli; Bacillales; Paenibacillaceae) | 0.00% | 1.49% | 0.000 | **0.000** |

*: The *p*-values were FDR-corrected to control for multiple testing.

**Supplemental Table 3.**

**The phylum and genus level of faecal samples microbiome compared between AC and CC children**

| **Phylum** | **AC** | **CC** | ***p*** | ***FDR_p**** |
| --- | --- | --- | --- | --- |
| Firmicutes | 49.42% | 40.85% | 0.004 | **0.008** |
| Bacteroidetes | 39.87% | 47.52% | 0.020 | **0.033** |
| Proteobacteria | 5.55% | 6.83% | 0.121 | 0.151 |
| Actinobacteria | 4.53% | 4.04% | 0.240 | 0.240 |
| Fusobacteria | 0.31% | 0.48% | 0.003 | **0.006** |
| Verrucomicrobia | 0.22% | 0.01% | 0.000 | **0.000** |
| Cyanobacteria | 0.05% | 0.17% | 0.165 | 0.183 |
| Saccharibacteria | 0.03% | 0.06% | 0.000 | **0.000** |
| Synergistetes | 0.01% | 0.00% | 0.034 | **0.049** |
| Deinococcus-Thermus | 0.00% | 0.03% | 0.000 | **0.000** |
| **Genus** |  |  |  |  |
| *Bacteroides*  (Bacteroidetes; Bacteroidia; Bacteroidales; Bacteroidaceae) | 27.37% | 33.85% | 0.059 | 0.085 |
| *Faecalibacterium*  (Firmicutes; Clostridia; Clostridiales; Ruminococcaceae) | 10.40% | 9.21% | 0.056 | 0.082 |
| *Lachnospiraceae UCG-008*  (Firmicutes; Clostridia; Clostridiales; Lachnospiraceae) | 5.27% | 5.40% | 0.550 | 0.582 |
| *Prevotella 9*  (Bacteroidetes; Bacteroidia; Bacteroidales; Prevotellaceae) | 4.69% | 7.18% | 0.086 | 0.119 |
| *Alistipes*  (Bacteroidetes; Bacteroidia; Bacteroidales; Rikenellaceae) | 4.57% | 2.81% | 0.000 | **0.001** |
| *Bifidobacterium*  (Actinobacteria; Actinobacteria; Bifidobacteriales; Bifidobacteriaceae) | 3.64% | 3.03% | 0.261 | 0.307 |
| *Sutterella*  (Proteobacteria; Betaproteobacteria; Burkholderiales; Alcaligenaceae) | 2.35% | 1.29% | 0.003 | **0.005** |
| *Ruminococcus 1*  (Firmicutes; Clostridia; Clostridiales; Ruminococcaceae) | 2.03% | 0.52% | 0.000 | **0.000** |
| *Subdoligranulum*  (Firmicutes; Clostridia; Clostridiales; Ruminococcaceae) | 2.03% | 1.68% | 0.045 | 0.069 |
| *Roseburia*  (Firmicutes; Clostridia; Clostridiales; Lachnospiraceae) | 1.75% | 1.91% | 0.825 | 0.843 |
| *Parabacteroides*  (Bacteroidetes; Bacteroidia; Bacteroidales; Porphyromonadaceae) | 1.64% | 2.00% | 0.185 | 0.234 |
| *Lachnospira*  (Firmicutes; Clostridia; Clostridiales; Lachnospiraceae) | 1.56% | 0.89% | 0.004 | **0.007** |
| *Ruminococcus 2*  (Firmicutes; Clostridia; Clostridiales; Ruminococcaceae) | 1.55% | 1.21% | 0.060 | 0.086 |
| *Blautia*  (Firmicutes; Clostridia; Clostridiales; Lachnospiraceae) | 1.51% | 2.32% | 0.011 | **0.019** |
| *Fusicatenibacter*  (Firmicutes; Clostridia; Clostridiales; Lachnospiraceae) | 1.33% | 1.68% | 0.063 | 0.089 |
| *Dialister*  (Firmicutes; Negativicutes; Selenomonadales; Veillonellaceae) | 1.29% | 1.84% | 0.477 | 0.516 |
| *Ruminococcaceae UCG-002*  (Firmicutes; Clostridia; Clostridiales; Ruminococcaceae) | 1.26% | 0.57% | 0.000 | **0.000** |
| *Parasutterella*  (Proteobacteria; Betaproteobacteria; Burkholderiales; Alcaligenaceae) | 1.25% | 2.38% | 0.000 | **0.000** |
| *[Eubacterium] eligens group*  (Firmicutes; Clostridia; Clostridiales; Lachnospiraceae) | 1.24% | 0.36% | 0.000 | **0.000** |
| *[Eubacterium] coprostanoligenes group*  (Firmicutes; Clostridia; Clostridiales; Ruminococcaceae) | 1.23% | 0.57% | 0.000 | **0.000** |
| *Peptoclostridium*  (Firmicutes; Clostridia; Clostridiales; Peptostreptococcaceae) | 1.21% | 0.49% | 0.000 | **0.000** |
| *Haemophilus*  (Proteobacteria; Gammaproteobacteria; Pasteurellales; Pasteurellaceae) | 1.21% | 0.74% | 0.049 | 0.074 |
| *[Eubacterium] oxidoreducens group*  (Firmicutes; Clostridia; Clostridiales; Lachnospiraceae) | 1.06% | 0.49% | 0.001 | **0.002** |
| *Barnesiella*  (Bacteroidetes; Bacteroidia; Bacteroidales; Porphyromonadaceae) | 1.03% | 0.31% | 0.000 | **0.001** |
| *Escherichia-Shigella*  (Proteobacteria; Gammaproteobacteria; Enterobacteriales; Enterobacteriaceae) | 0.17% | 1.13% | 0.000 | **0.000** |

*: The *p*-values were FDR-corrected to control for multiple testing.

**Supplemental Table 4.**

Phenotypes of bacteria function comparison between AC and CC children in OP and faecal samples

|  | **AC** | **CC** | ***FDR_p**** |
| --- | --- | --- | --- |
| **OP samples** |  |  |  |
| Gram Positive | 0.64±0.10 | 0.45±0.19 | **0.000** |
| Gram Negative | 0.36±0.10 | 0.55±0.19 | **0.000** |
| Oxygen Utilizing |  |  |  |
| Aerobic | 0.31±0.13 | 0.39±0.22 | 0.330 |
| Anaerobic | 0.31±0.15 | 0.23±0.16 | **0.016** |
| Facultatively anaerobic | 0.21±0.07 | 0.11±0.05 | **0.000** |
| Biofilm Forming | 0.41±0.13 | 0.36±0.20 | 0.120 |
| Pathogenic Potential | 0.92±0.07 | 0.80±0.23 | **0.002** |
| Mobile Element Containing | 0.50±0.09 | 0.51±0.22 | 0.907 |
| Oxidative Stress Tolerant | 0.18±0.11 | 0.22±0.16 | 0.445 |
| **Faecal samples** |  |  |  |
| Gram Positive | 0.70±0.16 | 0.64±0.12 | **0.012** |
| Gram Negative | 0.30±0.16 | 0.36±0.12 | **0.012** |
| Oxygen Utilizing |  |  |  |
| Aerobic | 0.03±0.03 | 0.03±0.02 | 0.515 |
| Anaerobic | 0.93±0.04 | 0.92±0.05 | 0.968 |
| Facultatively anaerobic | 0.02±0.01 | 0.02±0.02 | **0.041** |
| Biofilm Forming | 0.09±0.05 | 0.10±0.06 | 0.974 |
| Pathogenic Potential | 0.52±0.13 | 0.51±0.12 | 0.823 |
| Mobile Element Containing | 0.78±0.13 | 0.73±0.11 | **0.011** |
| Oxidative Stress Tolerant | 0.04±0.03 | 0.04±0.03 | 0.172 |

Bugbase tool was used to predict the organism level of microbiome phenotypes.

*: The *p*-values were FDR-corrected to control for multiple testing.

**Supplemental Table 5**

**Binomial probability test to determine consistent differences in distinct taxa between AC and CC children and different allergic conditions in AC children**

|  | significance level: 0.05 * | | | | | | significance level: 0.01 * | | | | | |
| --- | --- | --- | --- | --- | --- | --- | --- | --- | --- | --- | --- | --- |
|  | Mean difference #: 0.01% | | | Mean difference #: 0.1% | | | Mean difference #: 0.01% | | | Mean difference #: 0.1% | | |
| **OP** | N | n(%) | *P*^$^ | N | n(%) | *P*^$^ | N | n(%) | *P*^$^ | N | n(%) | *P*^$^ |
| Atopy | 69 | 42(60.8) | 0.091 | 39 | 22(56.4) | 0.522 | 42 | 27(64.3) | 0.088 | 24 | 17(70.8) | 0.064 |
| Food allergy | 75 | 63(84.0) | **0.000** | 43 | 36(83.7) | **0.000** | 49 | 39(79.6) | **0.000** | 30 | 23(76.7) | **0.005** |
| Wheezing | 76 | 42(55.3) | 0.422 | 49 | 30(61.2) | 0.152 | 50 | 29(58.0) | 0.322 | 33 | 23(69.7) | **0.035** |
| **Faecal** | N | n(%) | *P*^$^ | N | n(%) | *P*^$^ | N | n(%) | *P*^$^ | N | n(%) | *P*^$^ |
| Atopy | 59 | 36(61.0) | 0.117 | 30 | 21(70.0) | **0.043** | 39 | 22(56.4) | 0.522 | 17 | 12(70.6) | 0.143 |
| Food allergy | 55 | 13(23.6) | **0.000** | 33 | 4(12.1) | **0.000** | 37 | 8(21.6) | **0.001** | 21 | 2(9.5) | **0.000** |
| Wheezing | 47 | 14(29.8) | **0.008** | 36 | 5(13.9) | **0.000** | 30 | 8(26.7) | **0.016** | 24 | 2(8.3) | **0.000** |

*: selected taxa with significant differences (at two significance levels: *p*<0.05 and *p*<0.01) in relative abundance between AC and CC children at five taxonomic levels (phylum, class, order, family, and genus)

#: mean difference of taxa abundance between allergic condition positives and negatives was calculated, and those with mean difference over 0.01% or 0.1% were selected.

$: Binomial probability test was used to evaluate the consistent trend.
